# Novel Thyroid Hormone Receptor-β Agonist TG68 Exerts Anti-Inflammatory, Lipid-Lowering and Anxiolytic Effects in a High-Fat Diet (HFD) Mouse Model of Obesity

**DOI:** 10.3390/cells14080580

**Published:** 2025-04-11

**Authors:** Beatrice Polini, Caterina Ricardi, Francesca Di Lupo, Massimiliano Runfola, Andrea Bacci, Simona Rapposelli, Ranieri Bizzarri, Marco Scalese, Federica Saponaro, Grazia Chiellini

**Affiliations:** 1 Department of Surgical, Medical and Molecular Pathology and Critical Care Medicine, University of Pisa, Via Roma 56, 56126 Pisa, Italy; beatrice.polini@unipi.it (B.P.); c.ricardi@student.unisi.it (C.R.); f.dilupo1@studenti.unipi.it (F.D.L.); ranieri.bizzarri@unipi.it (R.B.); 2 Department of Pharmacy, University of Pisa, Via Bonanno 6, 56126 Pisa, Italy; massimiliano.runfola@pharm.ox.ac.uk (M.R.); andrea.bacci@phd.unipi.it (A.B.); simona.rapposelli@unipi.it (S.R.); 3 Institute of Clinical Physiology, Italian National Research Council, 56124 Pisa, Italy; marco.scalese@ifc.cnr.it

**Keywords:** obesity, microglia, neuroinflammation, neurodegeneration, anxiety, thyromimetics

## Abstract

Recent advances in drug development allowed for the identification of THRβ-selective thyromimetic TG68 as a very promising lipid lowering and anti-amyloid agent. In the current study, we first investigated the neuroprotective effects of TG68 on in vitro human models of neuroinflammation and β-amyloid neurotoxicity in order to expand our knowledge of the therapeutic potential of this novel thyromimetic. Subsequently, we examined metabolic and inflammatory profiles, along with cognitive changes, using a high-fat diet (HFD) mouse model of obesity. Our data demonstrated that TG68 was able to prevent either LPS/TNFα-induced inflammatory response or β-amyloid-induced cytotoxicity in human microglial (HMC3) cells. Next, we demonstrated that in HFD-fed mice, treatment with TG68 (10 mg/kg/day; 2 weeks) significantly reduced anxiety-like behavior in stretch–attend posture (SAP) tests while producing a 12% BW loss and a significant decrease in blood glucose and lipid levels. Notably, these data highlight a close relationship between improved serum metabolic parameters and a reduction of anxious behavior. Moreover, TG68 administration was observed to efficiently counteract HFD-altered central and peripheral expressions in mice with selected biomarkers of metabolic dysfunction, inflammation, and neurotoxicity, revealing promising neuroprotective effects. In conclusion, our work provides preliminary evidence that TG68 may represent a novel therapeutic opportunity for the treatment of interlinked diseases such as obesity and neurodegenerative diseases.

## 1. Introduction

Over the past two decades, it has become widely recognized that metabolic deregulations induced by a high fat diet (HFD) generate inflammation in peripheral tissues, which promotes the development of neuroinflammation. This inflammatory state can disrupt neuronal homeostasis, leading to the alteration of synaptic plasticity, memory disorders, and behavioral deterioration [1,2]. In addition to inflammation, mechanisms linking metabolic dysfunction and neurodegeneration include insulin resistance, immune response, oxidative stress, and mitochondrial dysfunction [3,4,5]. Notably, these pathological events cause a self-amplifying vicious cycle, ultimately leading to neuronal death and promoting the occurrence of neurodegenerative diseases [6]. Recent evidence suggest that in obese insulin-resistant subjects, careful attention to thyroid hormone homeostasis can prevent the worsening of metabolic conditions and the development or progression of cognitive decline [7]. Thyroid hormone (TH) plays a crucial role in regulating different physiological functions, including growth, metabolism, nervous system development, cardiac function, bone regulation, and adipose tissue function [8]. Alteration of the TH level impacts all metabolically active tissues, leading to different clinical conditions, such as obesity, diabetes, inflammatory liver steatosis, cardiovascular diseases, dementia, cognitive impairment, cancer, etc. In addition to their systemic effects, THs are essential for maintaining brain homeostasis, which prevents the development of neuroinflammation and, potentially, cognitive impairment [9]. It is known that the hippocampus, a key structure involved in spatial learning and memory, is very sensitive to the actions of THs due to the high expression of their receptors in this brain region [10]. Studies on hypothyroidism have reported an impairment of hippocampal glial function [11,12], oxidative responses [13], synaptic plasticity [14], and memory [15]. Notably, TH supplementation in hypothyroid animals has been shown to restore memory function and decrease neuroinflammatory markers [12,13,16].

By promoting an anti-inflammatory microglial phenotype, THs support tissue repair and neuroprotection. Microglia, the primary immune cells of the central nervous system, are crucial in maintaining brain homeostasis and responding to injury. However, dysregulated microglial activity has been implicated in neurodegenerative diseases such as Alzheimer’s and multiple sclerosis, where excessive activation contributes to inflammation and neuronal damage. The established link between Alzheimer’s pathology and hypothyroidism further underscores the importance of THs in mitigating neuroinflammation and preventing neurodegeneration [17].

**Figure 1:**
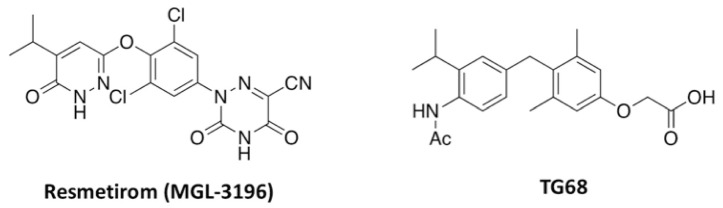
Novel Synthetic Thyroid Hormone Receptor-β Agonists. Chemical structures of resmetirom, the first synthetic thyromimetic approved for clinical use, and TG68.

Most TH effects are mediated by nuclear receptors (THRs), thyroid hormone receptor α (THRα) and thyroid hormone receptor β (THRβ), whose distribution is heterogeneous among different tissues and/or during developmental stages [18]. THRα isoform is predominantly in the heart, whereas expression of THRβ isoform is paramount in the liver, kidney, etc., which suggests higher therapeutic efficacy of tissue/isoform-specific modulators [19,20]. Indeed, in the last few decades, the drug-targetable property of THRs has been utilized to develop different THRβ selective modulators (Figure 1) to treat primarily lipid-related metabolic disorders, including dyslipidemia and non-alcoholic fatty liver disease/non-alcoholic steatohepatitis (NAFLD/NASH) [19,20,21,22,23,24,25,26,27]. Among them, resmetirom (Figure 1) was recently approved by the U.S. FDA, under the brand name Rezdiffra™, for treating patients with NASH [28,29], which provides the basis for research and development of new drugs for liver diseases. TG68 (Figure 1) is a recently developed THRβ selective thyromimetic produced by our group as a pro-drug of IS25 [24], a novel halogen-free THRβ selective agonist based on the scaffold of sobetirome [30], which has been found to share resmetirom’s capacity to strongly reduce liver fat accumulation in a diet-induced mouse model of NAFLD, thus revealing a good safety profile for long-term therapies [25]. In addition, recent in vitro studies have revealed that TG68 holds promise for neuroprotection and displays a remarkable ability to stimulate oligodendrocytes (OPC) differentiation and overcome inflammation-mediated pathological conditions [31], as well as to potently inhibit transthyretin (TTR)-mediated amyloidosis (TTRA) [32].

Taken together, all these findings suggest that TG68 may represent a promising multitarget agent for the treatment of interlinked diseases such as obesity and neurodegeneration. With the aim to expand our knowledge on the pleiotropic actions of this novel thyromimetic, we first explored TG68 neuroprotective effects on in vitro human models of neuroinflammation and β-amyloid neurotoxicity in the present work. Subsequently, we performed in vivo studies on a well-established model of obesity/neuroinflammation, namely the High Fat Diet (HFD) obese/insulin-resistant mouse model, to investigate the effects of chronic treatment with TG68 on energetic metabolism, neuroinflammation, and behavior. Furthermore, we try to ascertain the existence of a correlation between the observed metabolic and behavioral outcomes.

## 2. Materials and Methods

### 2.1. In Vitro Study

#### 2.1.1. Cell Cultures and Reagents

The human microglial clone 3 cell line (HMC3) (ATCC^®^ CRL-3304TM, Manassas, VA, USA) was maintained in high-glucose DMEM (D5796, Sigma-Aldrich, Milan, Italy) supplemented with 10% fetal bovine serum (F2442, Sigma-Aldrich, Milan, Italy) and a 1:1 mixture of streptomycin and penicillin (P4458, Sigma-Aldrich, Milan, Italy). Reagents used in the experiments included lipopolysaccharide (LPS) from *Escherichia coli* 0111:B4 (LPS25, Sigma-Aldrich, Milan, Italy), tumor necrosis factor-alpha (TNFα) (H8916, Sigma-Aldrich, Milan, Italy), and amyloid beta-peptide 25–35 (Aβ25–35) (A4559, Sigma-Aldrich, Milan, Italy). TG68 aliquots were stored at −20 °C in DMSO (D2650, Sigma-Aldrich, Milan, Italy) as a 25 mM stock solution and diluted to the desired final concentration in culture media.

#### 2.1.2. MTT Assay for Cell Viability

To assess cell viability, HMC3 cells were treated with increasing concentrations of TG68 (0.1–10 μM) and incubated at 37 °C for 24 h. The MTT reagent (3-(4,5-dimethylthiazol-2-yl)-2,5-diphenyltetrazolium bromide) (475989, Sigma-Aldrich, Milan, Italy) was then added at a final concentration of 0.5 mg/mL, and cells were incubated for an additional 3 h at 37 °C.

After removing 25 µL of medium from each well, 50 µL of DMSO was added to dissolve the formazan crystals. Absorbance was measured at 540 nm using a BIO-TEK microplate reader (Winooski, VT, USA). Cell viability was calculated relative to vehicle-treated controls.

The same experimental approach was applied to investigate the cytotoxic effects of Aβ25–35 (10 μM) on HMC3 cells over 24 in the presence or absence of varying concentrations of TG68 (0.1–10 μM).

#### 2.1.3. Inflammatory Response in LPS/TNFα-Treated HMC3 Cells

The levels of IL-6 and IL-10 were quantified using specific ELISA kits (RAB0306 for IL-6 and RAB0244 for IL-10, Sigma-Aldrich, Milan, Italy). HMC3 cells were pretreated with TG68 (0.1–10 μM) for 1 h, followed by exposure to LPS (10 μg/mL) and TNFα (50 ng/mL) for 24 h. Vehicle-treated cells served as controls for comparison.

#### 2.1.4. Inflammatory Response in HMC3 Cells Exposed to Aβ25–35

The inflammatory response triggered by Aβ25–35 (10 μM, 24 h) in HMC3 cells was evaluated using ELISA kits. In addition to measuring IL-6 and IL-10, TNFα (RAB1089, Sigma-Aldrich, Milan, Italy) and NF-κB (85-86082-11, Thermo Fisher Scientific, Carlsbad, CA, USA) levels were assessed according to the manufacturer’s protocols.

Before Aβ25–35 exposure, HMC3 cells were pretreated with varying concentrations of TG68 for 1 h. Vehicle-treated cells served as controls for baseline measurements.

### 2.2. In Vivo Study

#### 2.2.1. Animals

Forty-five male CD1 mice (Charles River Laboratories, France) were divided into two groups, namely n = 30 fed with ad libitum High Fat Diet (HFD) and n = 15 fed with Standard Diet (SD). HFD provides 18.3% kcal from proteins, 21.4% kcal from carbohydrates, and 60.8% kcal from lipids (5.1 kcal/g: arachidic acid C-20:0, Eicosanoid acid C-20:1, α-Linolenic acid C-18:3, Palmitic acid C-16:0, Stearic acid C-18:0, Oleic acid C-18:1), compared to SD providing 24% kcal from proteins, 58% kcal from carbohydrates and 18% from lipids (3.1 kcal/g) (Kros.way srls, Monza (MB), Italy). These conditions were maintained for 10 weeks to achieve obesity and insulin-resistance condition (1), and subsequently, HFD group was split into two groups: n = 15 HFD mice treated with TG68 (10 mg/kg/day; for 14 days) in drinking water, and n = 15 HFD mice not exposed to drug treatment.

All mice were kept in the same colony room at constant temperature (22 °C) and humidity (50%) on a reversed 12 h light/dark cycle (lights on at 1800 h, off at 0600 h), which was used to perform experiments during the dark cycle when the mice had the highest activity.

Mice development was monitored by weighing the animals once a week. The time frame for habituation to handling consisted of 2 min sessions of animal handling every two days, from the day of their arrival at the animal facility throughout the duration of the experiments. Additionally, in the week leading up to the behavioral tests, handling was conducted daily, and the animals were acclimated to the behavioral testing room. After 2 weeks of treatment or placebo behavioral tests have been performed. Subsequently, all mice were then sacrificed, and blood and tissues were collected and stored at −80 °C for subsequent analyses.

All the experiments were conducted in compliance with the European Community Council Directive of 24 November 1986 (86/609/EEC) and in compliance with L.D. 4 March 2014 No. 26 for minimizing animal suffering and to use only the number of animals necessary to produce reliable results. The Italian Ministry of Health approved the use of animals in this protocol (approval number: 260/2019PR; A039F.4).

#### 2.2.2. Biochemical Testing and Tissues Analysis

- Analysis of serum triglycerides, cholesterol, and glucose

The blood collected during sacrifice underwent centrifugation at 1000× *g* for 15 min at 4 °C. The serum (plasma) obtained was utilized to quantify and determine the circulating concentration of selected metabolic markers, such as glucose (Optium Neo Glucometer, FreeStyle, Witney, Oxon, UK), tryglicerides (MAK564, Trygliceride Quantification kit, Sigma-Aldrich) and cholesterol (Cholesterol Quantification Assay kit, Sigma-Aldrich).

- Gene expression analysis

To perform gene expression analysis, total RNA was isolated from various tissues (i.e., hypothalamus, liver, and adipose tissue) using the RNeasy Mini Kit (74104, Qiagen, Hilden, Germany), and 1 μg of extracted RNA was reverse transcribed into first-strand cDNA using the iScript™ gDNA Clear cDNA Synthesis Kit (1725035, Bio-Rad, Milan, Italy), following protocols provided by the manufacturers. The relative expression levels of target genes were quantified by real-time PCR using SYBR Green dye and the CFX Connect Real-Time PCR Detection System (Bio-Rad, Milan, Italy).

The thermal cycling conditions included an initial denaturation step at 95 °C for 30 s, followed by 40 cycles of denaturation at 95 °C for 5 s, and annealing/extension at 60 °C for 15 s. A melting curve analysis was conducted from 65 °C to 95 °C with 0.5 °C increments every 5 s to confirm the specificity of the amplification products and detect potential primer dimers. Primers were designed using Beacon Designer Software (version 8.0, Premier Biosoft International, Palo Alto, CA, USA), employing a junction primer strategy whenever feasible (Table 1). Additionally, a negative control for reverse transcription was included to rule out contamination from residual genomic DNA. Gene expression data were analyzed using the 2^−ΔΔCt^ method and expressed as fold change relative to the corresponding control.

- Inflammatory Response in HFD mice

The systemic inflammatory response induced by HFD was evaluated using specific ELISA assays. The levels of pro-inflammatory cytokines TNFa (RAB0477, Sigma-Aldrich) and IL6 (RAB0308, Sigma-Aldrich) were evaluated in available plasma samples, while Brain Derived Neurotrophic Factor (BDNF) was analyzed in the hypothalamus (BSENBEK-2211-1P, VWR srl).

#### 2.2.3. Behavioral Testing

On the day of the tests, the mice were transferred to the testing room at least 60 min before the beginning of the experiments. Mice behavior was monitored and recorded through an overhead camera positioned above the Open Field (OF) and Elevated Plus Maze (EPM) apparatus. The videos generated during the EPM were scored manually in a blind fashion by three independent observers. The videos generated during OF were analyzed by dedicated software (SMART.3.0, Panlab, Cornella, Barcelona).

Open Field (OF): the standard open field test is commonly used to assess locomotor, exploratory, anxiety-like behaviors, behavioral responses to novelty in laboratory animals, and depressive behaviors [33]. The open field test examines the natural opposite tendencies to explore a novel environment and the tendency to avoid a brightly lit area at the center of the field open area. When anxious, rodents tend to avoid discovery and stay put or move along the walls (thigmotaxis).

Each mouse was placed at the center of the squared open arena (90 cm × 90 cm) for 10 min, and its behavior was video-recorded, as previously described [34].

Elevated Plus Maze (EPM): the EPM apparatus consisted of 4 arms (25 × 5 cm) forming a plus sign, elevated 50 cm above the floor, with two open and two closed walls. Each mouse was allowed to freely explore the maze for 5 min. The EPM measures the conflict between two opposite natural instincts of exploring or avoiding open, unprotected spaces. The higher the “anxiety” levels, the lower the proportion of explorations of open spaces (open arms) versus dark spaces (closed arms). Increased time spent in open arms indicates a lower degree of “anxiety” in the animals. At the end of each individual trial, the maze was cleaned with 15% ethanol solution to remove olfactory cues. Videos were recorded of the 5 min EPM for each mouse and subsequently analyzed in a blind fashion by three independent observers and compared. Time spent in open arms and in closed arms were manually scored and the following behavioral acts were measured: (i) SAP (stretch–attend posture): standing still, with the body elongated; (ii) F-SAP (forward in SAP): moving forward with elongated body; (iii) HOLES: sniffing at the holes or dipping the snout into them; (iv) HEAD-UP: raising of the head; (v) GO: going forward and backward; (vi) IMMOBILITY: crouch/freeze.

### 2.3. Statistical Analysis

Statistical analyses were performed using GraphPad Prism version 9.0 for Mac (Graph-Pad Software, San Diego, CA, USA). Significant differences among different treatments were calculated using one-way ANOVA followed by Dunnett’s or Tukey’s multiple comparisons test. Associations between serum metabolic marker levels and behavioral test parameters were analyzed using the Spearman correlation coefficient.

Data are reported as mean ± SEM. Differences at *p* < 0.05 were considered statistically significant.

## 3. Results

### 3.1. In Vitro Experiments

#### TG68 Prevents LPS/TNFα and Aβ-Induced Inflammatory Activation in HMC3 Cells

**Figure 2:**
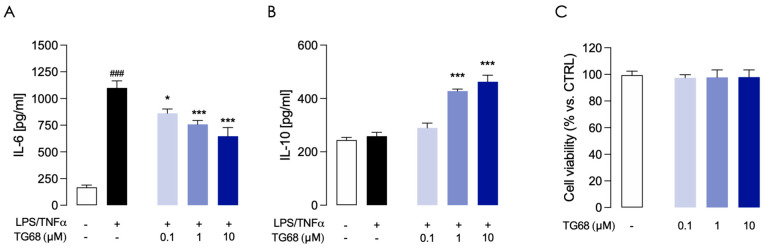
Effects of pretreatment with TG68 on inflammatory response in LPS/TNF⍺-stimulated HMC3 cells. Selected concentrations of TG68 (0.1, 1, and 10 µM) decreased the levels of pro-inflammatory interleukin IL-6 (**A**), induced the release of anti-inflammatory interleukin IL-10 (**B**), and did not interfere with cell viability (**C**). Data represent means ± SEM from three independent experiments performed in duplicate. Statistical analysis was performed by ordinary one-way ANOVA followed by Dunnett’s multiple comparisons test. * *p* < 0.05 and *** *p* < 0.005 compared to LPS/TNF⍺ treated cells; ### *p* < 0.005 compared to vehicle-treated cells used as control.

Microglia, the innate immune responders of the central nervous system, are key mediators of neuroinflammation in neurodegenerative diseases (NDDs) [35]. It is widely known that in vitro treatment of microglial cells with pro-inflammatory stimuli such as LPS and TNFα induces M1 polarization characterized by the production of high levels of pro-inflammatory cytokines [36]. Therefore, we first examined whether TG68 exerts an anti-inflammatory effect on microglia. In dose-response experiments, pretreatment of human microglia cells (HMC3) with TG68 (0.1, 1, and 10 μM), followed by inflammatory stimulation with LPS (10 μg/mL)/TNFα (50 ng/mL) for 24 h, resulted in a significant (*p* < 0.05) decrease in pro-inflammatory IL-6 (Figure 2A), and a significant increase in anti-inflammatory IL-10 (Figure 2B), highlighting anti-inflammatory activity for TG68. The total absence of cytotoxicity for TG68 at all tested doses was confirmed in HMC3 cells through cell viability assays (MTT) (Figure 2C).

**Figure 3:**
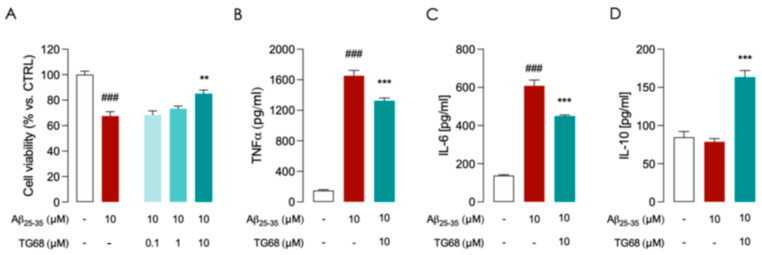
Effects of TG68 on Aβ_25–35_ treated HMC3 cells. HMC3 cells were treated with 10 µM Aβ_25–35_ for 24 h in the presence of different concentrations of TG68 observing a dose-dependent cytoprotective effect (**A**). Aβ_25-35_ exposure induced an increased release of pro-inflammatory cytokines TNF-α (**B**) and IL-6 (**C**), with no effect on the release of anti-inflammatory IL-10 (**D**). The most effective TG68 concentration (10 µM) was also able to reduce the levels of pro-inflammatory cytokines IL-6 (**B**) and TNF-⍺ (**C**) and to induce the release of the anti-inflammatory IL-10 (**D**). Data represent means ± SEM from three independent experiments performed in duplicate. Statistical analysis was performed by ordinary one-way ANOVA followed by Dunnett’s multiple comparisons test. ** *p* < 0.01 and *** *p* < 0.005 compared to Aβ25–35 exposed cells; ### *p* < 0.005 compared to vehicle-treated cells used as control.

It is well known that β-amyloid oligomers can activate microglia to secrete pro-inflammatory factors that cause neurotoxicity [37]. Blocking the vicious cycle among Aβ peptide accumulation, activated microglia, and pro-inflammatory factors can be crucial for halting neurodegeneration in AD [38]. In our experimental settings, exposure of HMC3 cells to 10 μM Aβ_25–35_ for 24 h led to a significant (*p* < 0.05) cytotoxic effect (Figure 3A) associated to increased release of both TNF-α and IL-6 (Figure 3B,C). In Aβ-treated HMC3 cells, the pretreatment with 10 μM TG68 for 24 h was observed to significantly halt Aβ-cytotoxicity (Figure 3A) and to reduce the production of TNF-α and IL-6 (Figure 3B,C). An increase in anti-inflammatory cytokine IL-10 levels was also observed after pretreatment with 10 μM TG68 (Figure 3D). Taken together, these findings suggest the potential of TG68 to prevent Aβ-induced cytotoxicity and microglia activation.

### 3.2. In Vivo Experiments

#### 3.2.1. In Diet-Induced Obese Mice TG68 Treatment Causes a Significant Reduction in Body Weight (BW) and Blood Lipids and Glucose Levels

High-fat diet (HFD) consumption is considered one of the main factors for obesity induction and its association with metabolic and neurological diseases in humans [39]. High-fat diet (HFD) feeding can cause imbalances in the adipose tissue (AT) environment and alter its anti-inflammatory state by recruiting pro-inflammatory immune cells; a chronic inflammatory state may be achieved, both local and systemic. Therefore, a better understanding of the interplay between obesity and chronic inflammation may be crucial for the treatment of obesity.

Previous studies demonstrated that TG68 could be safely administered for up to 14 days at the dose of 10 mg/kg/day to a mouse model of non-alcoholic fatty liver disease (NAFLD) [25]. For this reason, in our experiments we administered TG68 at the same dose (10 mg/kg/day; 2 weeks) to mice fed an HFD for 12 weeks.

**Figure 4:**
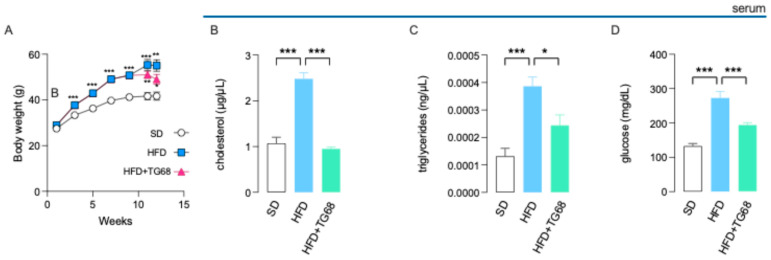
In HFD mice, TG68 induces a significant body weight loss and modulates the circulating levels of metabolic parameters. Administration of TG68 (10 mg/kg/day) for two weeks to HFD mice (10 weeks) led to 12% body-weight loss (**A**) and a significant decrease in serum cholesterol, triglycerides, and glucose (**B**–**D**). Each assay was carried out in technical duplicates, and values represent the mean ± SEM. Statistical analysis was performed by ordinary one-way ANOVA followed by Tukey’s (**A**) or Dunnett’s (**B**–**D**) multiple comparisons test (* *p* < 0.05, ** *p* < 0.01, *** *p* < 0.005).

**Figure 5:**
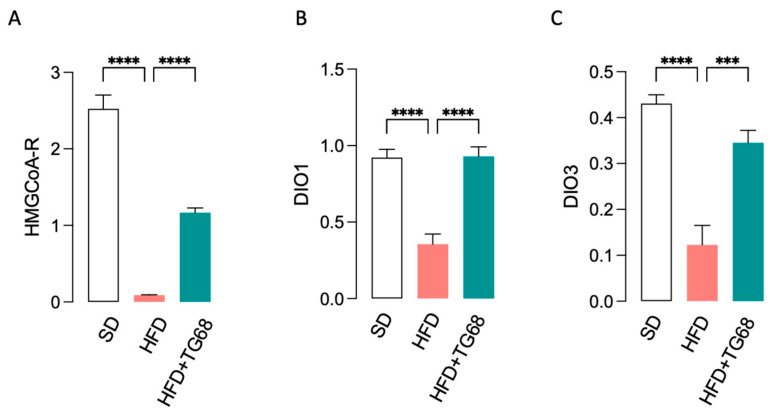
Effect of TG68 on transcriptional expression of hepatic genes. qPCR analysis revealed that TG68 counteracted HFD-induced changes in the hepatic expression of β-hydroxy-β-methylglutaryl-CoA (*HMG-CoA*) reductase (**A**) and of THRβ -target genes, namely *DIO1* (**B**) and *DIO3* (**C**). Each experiment was performed in technical triplicate, and values represent the mean ± SEM. Statistical analysis was performed by ordinary one-way ANOVA followed by Dunnett’s multiple comparisons test (*** *p* < 0.005; **** *p* < 0.001).

In HFD mice chronic treatment with TG68 (10 mg/kg/day; 2 weeks; in drinking water) produced a 12% BW loss (Figure 4A) and a significant (*p* < 0.05) decrease in blood lipids and glucose levels (Figure 4B–D). Since the treatment of HFD-fed mice with 10 mg/kg of TG68 for two weeks led to a relevant reduction in serum cholesterol level (Figure 4B), we further analyzed by qPCR the mRNA levels of β-hydroxy-β-methylglutaryl-CoA (*HMG-CoA*) reductase, the rate-limiting enzyme for cholesterol synthesis [40], on liver samples of all treatment groups. In addition, to confirm that the effect of TG68 was associated with THRβ activation, we also assessed the expression of THRβ -target genes, such as *DIO1* and *DIO3*. As shown in Figure 5, while HFD caused decreased expression of all the THRβ -target genes examined in the liver, the expression of *HMG-CoA*-reductase, *DIO1*, and *DIO3* was significantly increased following treatment with TG68 for two weeks.

#### 3.2.2. TG68 Counteracts Obesity-Induced Transcriptional Changes in Adipose Tissue and Hypothalamus

**Figure 6:**
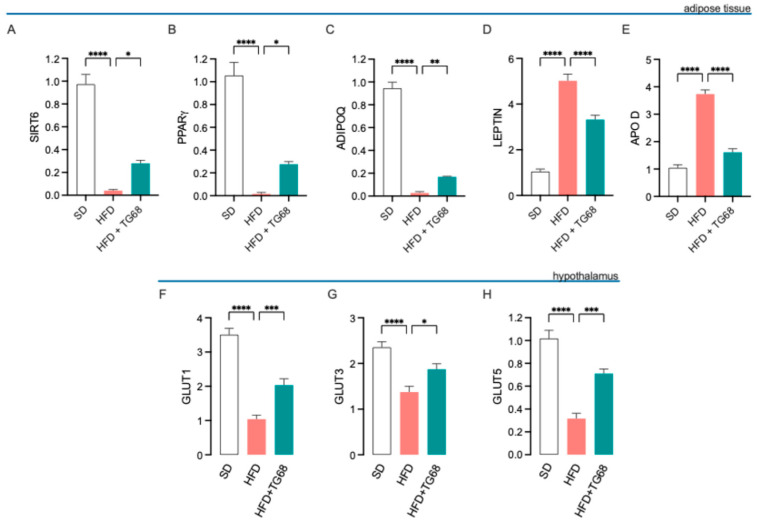
Effects of TG68 on transcriptional expression of metabolism-related genes in adipose tissue and hypothalamus. qPCR analysis revealed that TG68 counteracted HFD-induced changes in the expression of genes that regulate lipid metabolism and mobilization, glucose uptake, and metabolism. Specifically, TG68 treatment increased the expression of *SIRT6* (**A**), *PPARγ* (**B**), and *ADIPOQ* (**C**), while decreasing leptin (**D**) and *APOD* (**E**) expression in adipose tissue. Additionally, in the HFD+TG68 group, increased expression of hypothalamic glucose transporters, namely *GLUT1*, *GLUT3*, and *GLUT5*, was observed compared to HFD mice (**F**–**H**). Each experiment was performed in technical triplicate, and values represent the mean ± SEM. Statistical analysis was performed by ordinary one-way ANOVA followed by Dunnett’s multiple comparisons test (* *p* < 0.05, ** *p* < 0.01; *** *p* < 0.001; **** *p* < 0.0001).

Due to the observed significant differences in circulating metabolic markers, we performed qPCR on adipose tissue (AT) and hypothalamus to assess whether TG68 administration could counteract HFD-induced alteration of the expression of key metabolic markers. Our analysis revealed that TG68 increased the expression of *SIRT6*, *PPARγ,* and *ADIPOQ* expression in AT (Figure 6A–C) while inducing a decrease in *Leptin* and *APOD* expression (Figure 6D,E). Furthermore, *GLUT1*, *GLUT3*, and *GLUT5* expression increased in the hypothalamus following TG68 treatment (Figure 6F–H).

#### 3.2.3. TG68 Limits Systemic Inflammation and Attenuates the Transcriptional Changes Induced by HFD on the Hypothalamus of Obese Mice

It is extensively documented that obesity is a condition of chronic low-grade inflammation associated with an increased secretion of adipokines, chemokines, and cytokines, including TNFa and IL-6, from AT. Peripheral inflammation can extend to the brain, influencing critical metabolic regulatory regions and leading to neuroinflammation and neurodegeneration.

**Figure 7:**
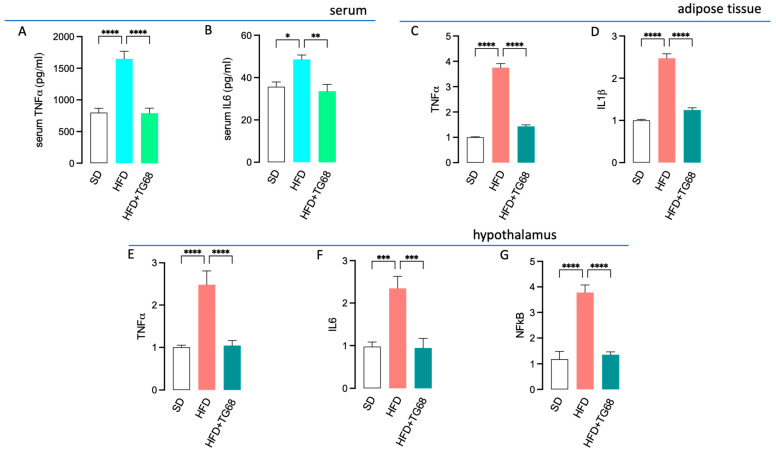
Chronic treatment with TG68 counteracts the inflammatory response in HFD mice. Chronic treatment with TG68 (10 mg/kg/die) significantly reduced the serum levels of pro-inflammatory cytokines TNFα (**A**) and IL-6 (**B**) in HFD mice. Accordingly, the HFD-induced increased expression of *TNFα* (**C**,**E**) in both adipose tissue and hypothalamus, as well as *IL-1β* (**D**) in adipose tissue and *IL6* (**F**) in hypothalamus, was totally abolished by TG68. In HFD mice hypothalamus, the significant increase in *NF-kB* expression (**G**) was effectively reversed by TG68 treatment. Each experiment was performed in technical triplicate, and values represent the mean ± SEM. Statistical analysis was performed by ordinary one-way ANOVA followed by Tukey’s test (* *p* < 0.05, ** *p* < 0.01; *** *p* < 0.001; **** *p* < 0.0001).

In mice, exposure to HFD for 12 weeks was observed to promote a marked inflammatory response, characterized by elevated serum levels of TNFα and IL6 (Figure 7A,B), as well as increased expression of *TNFα* and *IL1b* in the AT (Figure 7C,D) and *TNFα*, *IL6*, and *NF-kB* in the hypothalamus (Figure 7E–G). Notably, in the same mouse model of obesity, chronic administration of TG68 (10 mg/kg/day) significantly counteracted all the HFD-induced inflammatory responses (Figure 7).

**Figure 8:**
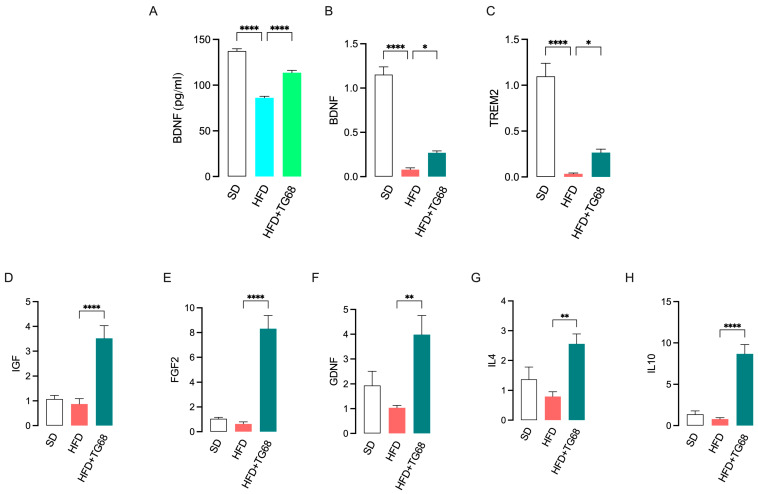
TG68 induces a neuroprotective effect on the hypothalamus of HFD mice. In HFD mice hypothalamus, TG68 counteracts a significant decrease in BDNF, either at protein (**A**) and transcriptional (**B**) levels. In hypothalamus of HFD mice, TG68 treatment increases the transcriptional expression of *TREM2* (**C**), *IGF1*(**D**), *FGF2* (**E**), *NGF* (**F**), IL4 (**G**), and *IL10* (**H**). Each experiment was performed in triplicate, and values represent the mean ± SEM. Statistical analysis was performed by ordinary one-way ANOVA followed by Tukey’s test; * *p* < 0.05, ** *p* < 0.01; **** *p* < 0.0001.

In addition, TG68 treatment was also observed to partially revert the reduced transcription and release of brain-derived neurotrophic factor (BDNF) detected in the hypothalamus of HFD mice (Figure 8A,B), thus suggesting a neuroprotective role for this novel thyromimetic.

Triggering receptor expressed on myeloid cells-2 (TREM2) is a cell surface receptor predominantly expressed in microglia of the central nervous system (CNS) that, when activated, initiates a signal transduction cascade that triggers a switch in these cells away from a pro-inflammatory phenotype to an anti-inflammatory, phagocytic, restorative phenotype [41]. Several studies have shown that TREM2 functional deficiency exacerbates neurodegenerative changes in inflammation-related diseases, including AD [42]. In addition, decreased central and peripheral expression of TREM2 has been detected in obese subjects [43]. Notably, TREM2 is a positively regulated TH target gene, and as recently reported by Scanlan et al. [44], T3 and novel TRβ-selective agonist Sob-AM2 stimulate *TREM2* expression in macrophages and microglia, paving the way for the future use of TREM2 signaling pathway as a pharmacological target of new thyromimetics. Consistently, we observed that TG68 administration partially reverted the reduced expression of *TREM2* detected in the hypothalamus of HFD mice (Figure 8C) while producing a significantly increased expression of gene markers of microglia anti-inflammatory and neuroprotective phenotype (M2) [5,45,46], including insulin-like growth factor (*IGF*), fibroblast growth factor (*FGF-2*), glial cell line-derived neurotrophic factor (*GDNF*), and anti-inflammatory cytokines *IL4* and *IL10* (Figure 8E–H), providing compelling evidence of TG68 anti-inflammatory and neuroprotective action.

#### 3.2.4. TG68 Improves Anxiety-like Behavior Induced by HFD

**Figure 9:**
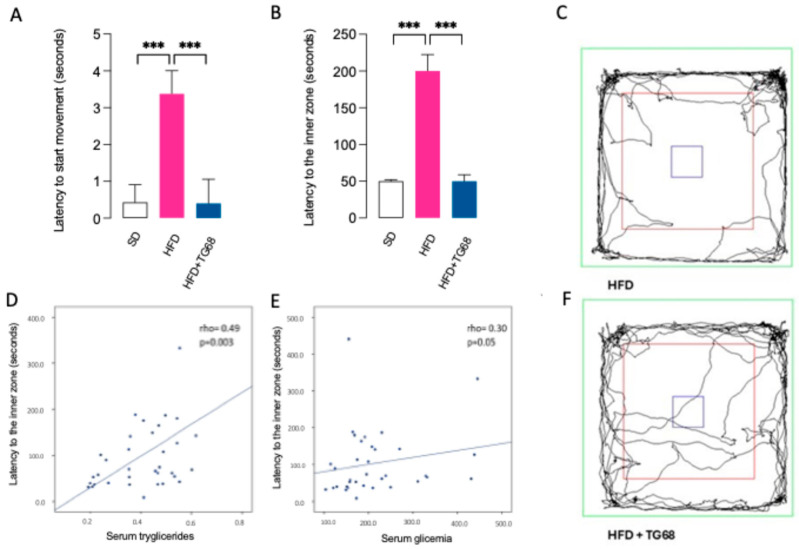
TG68 improves anxiety-like behavior at open field test (**A**,**B**). In HFD mice, TG68 significantly reduces the latency to start movement and latency to reach the inner zone of the arena, suggesting an improvement of anxiety-like behavior (SD: mice fed with standard diet n = 15, HFD: mice fed with HFD n = 15, HFD+TG68: mice fed with HFD and treated with TG68 n = 15). (**C**,**F**) Example trajectories for mice during open field test. (**D**,**E**) Latency to the inner zone is positively correlated with serum triglycerides and glucose (in the whole HFD group n = 30). Values represent the mean ± SEM. Statistical analysis was performed by ordinary one-way ANOVA followed by Tukey’s test; linear regression; *** *p* < 0.005.

Numerous lines of evidence suggest HFD facilitates the development of anxiety-related behavior, and a link to microglia activation and neuroinflammation has been proposed [47]. In light of the well-documented ability of TG68 to improve lipid metabolism and reduce neuroinflammation in HFD mice, we further extended our investigation to the evaluation of its anxiolytic ability. During an open field test (OFT) [48], we observed that obese mice showed significantly increased anxiety-like behavior (Figure 9A,B); indeed, when compared to SD-mice, HFD mice showed higher latency to start the movement and higher latency to reach the inner (center) zone of the arena, which are related to the tendency to avoid discovery and stay along the walls—thigmotaxis—of anxious animals [49]. This tendency was counteracted by TG68 treatment, which resulted in reducing the latency to start the movement and the latency to reach the center of the arena (Figure 9A–D).

Moreover, a positive correlation has been found in the whole group between latency to reach the inner zone of the arena and serum triglycerides (rho = 0.49, *p* = 0.003) and glucose (rho = 0.30; *p* = 0.04) (Figure 9E,F), suggesting that and the higher is hypertriglyceridemia and hyperglycemia, the worse is the performance at OFT.

**Figure 10:**
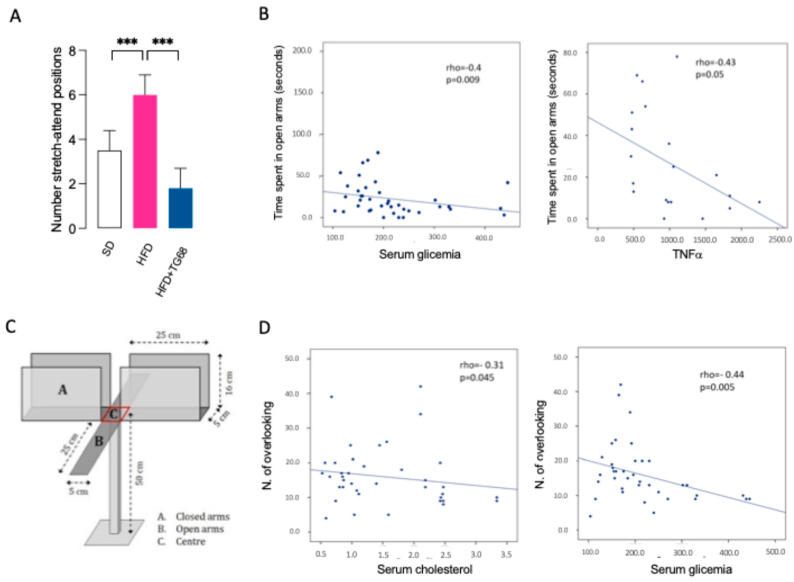
TG68 improves anxiety-like behavior at elevated plus maze test (**A**). In HFD mice, TG68 significantly reduces the SAP, suggesting an improvement of anxiety-like behavior (SD: mice fed with standard diet n = 15, HFD: mice fed with HFD n = 15, HFD+TG68: mice fed with HFD and treated with TG68 n = 15) (**B**). In the whole groups, there was an inverse correlation between the time spent in the open arms and serum glucose (rho = −0.40; *p* = 0.009, n = 35 available samples) and serum TNFα (rho = −0.43; *p* = 0.04, n = 20 available samples) (**C**). Inverse correlation between the total number of overlooking and serum cholesterol (rho = −0.31, *p* = 0.04, n = 35) and serum glucose (rho = −0.44, *p* = 0.005, n = 35) (**D**). Apparatus used for elevated plus maze test. Values represent the mean ± SEM. Statistical analysis was performed by ordinary one-way ANOVA followed by Tukey’s test; linear regression; *** *p* < 0.005.

To explore in more detail the potential anxiolytic effect of TG68, we performed an Elevated Plus Maze (EPM) test (Figure 10A–D) [50].

No significant differences were found among the three groups in the time spent in open/closed arms (*p* = 0.7). However, there was a significant difference among the groups in the numbers of stretch–attend positions (SAP, *p* = 0.010), which are related to internal exploratory conflict [49]. At post hoc analysis, HFD mice showed a higher number of SAP compared to SD-mice (*p* = 0.041) and TG68 treated mice (HFD+TG68 mice) (*p* = 0.004) (Figure 10A).

In the whole groups, at EPM, an inverse correlation has been found between the time spent in the open arms and serum glucose (rho = −0.40; *p* = 0.009) and serum TNFα (rho = −0.43; *p* = 0.04) (Figure 10B). An inverse correlation was noticed between the total number of overlooking and serum cholesterol (rho = −0.31, *p* = 0.04) and serum glucose (rho = −0.44, *p* = 0.005) (Figure 10C). These data suggest that better serum parameters are related to a less anxious behavior, namely exploring open arms and overlooking.

Collectively, our data suggest that TG68 administration might counteract HFD-induced metabolic and behavioral alterations.

## 4. Discussion

Obesity is widely recognized as a condition that not only affects peripheral tissues but also leads to significant neuroinflammation. This chronic low-grade inflammation originates in adipose tissue and is implicated in various cognitive deficits, including impairments in memory and learning. A large amount of scientific evidence supports the existence of at least two possible mechanisms underlying the linkage between HFD/obesity and neuropsychological diseases. First, HFD can increase microglial activation and neuroinflammatory responses, affecting synaptic density and leading to learning and cognitive deficits [51]. Second, HFD/obesity can promote the progression of neurodegenerative diseases in both humans and rodents via insulin resistance [52], further promoting mitochondrial dysfunction [53,54]. Notably, mitochondrial damage, neuroinflammation, and insulin resistance caused by HFD may form a mutually reinforcing vicious cycle, ultimately leading to the death of neurons and promoting the progression of neurodegenerative diseases [6].

Driving neuroinflammation involves blood-brain barrier (BBB) dysregulation and elevated levels of pro-inflammatory cytokines, such as TNF-α and IL-6, which are excessively released during obesity. The complex interplay between peripheral and neuroinflammatory processes is particularly evident in the hypothalamus, a brain region well-known to be affected by food habits and a key regulator of energy balance and metabolic homeostasis [55].

Hypothalamic inflammation is perpetuated by inflammatory and immune signals from peripheral sources, which activate microglial cells and astrocytes [56]. Subsequent release of neurotoxic substances can disrupt the integrity of the blood-brain barrier (BBB), which is already compromised due to systemic inflammation [57]. Prolonged consumption of a high-fat diet (HFD) may lead to inflammation, affecting brain regions outside the hypothalamus that are involved in mood regulation and anxiety, such as the prefrontal cortex, amygdala, and hippocampus [58]. As a result, obesity is often associated with a high prevalence of anxiety and mood disorders, besides being a risk factor for other comorbidities, such as insulin resistance, dyslipidemia, type 2 diabetes, cardiovascular diseases, and inflammation, collectively referred to as the metabolic syndrome [34]. The discovery of novel therapeutics for obesity management is essential for minimizing potential harms and optimizing overall health.

In this contest, THRβ-selective thyromimetic TG68 has been found to provide beneficial effects by mimicking thyroid hormone in its ability to reduce hepatic fat accumulation in the absence of unwanted toxic effects, mainly mediated by THRα, representing an attractive candidate for the treatment of primarily lipid-related metabolic disorders, including dyslipidemia and non-alcoholic fatty liver disease/non-alcoholic steatohepatitis (NAFLD/NASH) [24]. Recent data demonstrated the capability of TG68 to stimulate oligodendrocytes (OPC) differentiation and overcome inflammation-mediated pathological conditions [31], as well as to potently inhibit transthyretin (TTR)-mediated amyloidosis (TTRA) [32], suggesting a potential role in counteracting neuroinflammation and mitigating neuropsychological symptoms.

Our study supports a potential neuroprotective role for THRβ-selective thyroid hormone analog TG68, highlighting its ability to prevent inflammatory responses on in vitro models of microglial-mediated inflammation and to counteract central and peripheral alterations in the expression of several biomarkers of metabolic dysfunction, inflammation, and neurotoxicity, which are caused by HFD-induced obesity in mice, leading to BW loss, reduction in blood lipid, glucose, and pro-inflammatory cytokines levels, which are associated to a decrease in inflammation in both adipose tissue and hypothalamus. Indeed, TG68 seems able to enhance the expression of neurotrophic factors (i.e., *IGF*, *FGF*, *GDNF*, and *BDNF*), promoting a shift from the pro-inflammatory phenotype toward the anti-inflammatory M2 phenotype, thereby potentially ameliorating neuroinflammation associated with various neurological disorders. Collectively, all these findings underscore the dual role of TG68 in addressing peripheral metabolic derangements and central neuroinflammatory processes.

Animal models of HFD feeding have amply demonstrated how the inflammatory process associated with obesity may be involved in peripheral and brain disorders [39,59], and a diet rich in fats has been demonstrated to be linked to both metabolic dysfunction and neuropsychological or psychiatric disorders [60,61,62].

The current research firstly confirmed that in mice, a diet comprising 60% of caloric intake from fats (HFD) led to systemic inflammation, inducing a noteworthy effect on body composition, lipid metabolism, and glucose regulation. A second aspect of our experimental design revealed that exposure of mice to HFD-induced behavioral effects similar to those observed in chronic stress models of anxiety and stress [63,64]. These changes in behavior appear to be connected to heightened activation of inflammatory cytokines and disruptions in neurogenesis and neurotrophic signaling within the hippocampus and prefrontal cortex. Inflammatory cytokines such as IL-1β and TNF-α can access the brain via specific receptors located in the blood-brain barrier (BBB) or through passive routes via circumventricular organs. Additionally, damaged neurons and glial cells may release damage-associated molecular patterns (DAMPs), triggering a cascade of pro-inflammatory cytokines [65].

Thyroid hormones (THs) regulate neuroinflammatory processes and maintain brain homeostasis by modulating the expression of neurotrophic factors, such as BDNF, NGF, FGF, and GDNF. During inflammation, increased levels of pro-inflammatory cytokines (e.g., TNF-α, IL-6) disrupt the balance of neurotrophic factors, leading to impaired neuronal survival, synaptic plasticity, and tissue repair. This imbalance contributes to heightened anxiety and cognitive deficits, as reduced neurotrophic support and chronic inflammation synergistically affect brain regions involved in emotion regulation, such as the amygdala and prefrontal cortex. The interplay between THs, cytokines, and neurotrophic factors underscores their relevance as therapeutic targets for addressing neuroinflammation and anxiety.

Numerous studies have established that pro-inflammatory state within brain tissues plays a crucial role in cognitive impairment and the manifestation of anxiety and depression-related behaviors [66,67,68,69,70,71,72]. Targeting inflammation has been recently proposed as a potential therapeutic approach for treating anxiety-based disorders in the future [73]. However, more comprehensive research is still needed to thoroughly understand the mechanisms that lead to neuropsychological disorders following HFD consumption and obesity.

Moreover, we used a battery of behavioral tests to assess the potential action of TG68 on anxiety and stress response. The open field and elevated plus maze tests are clearly recognized in the literature to assess anxiety-like behavior, demonstrating high reliability, particularly when administered in conjunction [74]. A recent investigation indicated that the elevated plus maze (EPM) is less affected by environmental variables and age, while the open field (OF) test is comparatively less stressful for rodents [75]. In short, anxiety-related behaviors in animal models consist of a complex, multidimensional framework that includes both straightforward components and functional interactions, as highlighted by Carola et al. [76]. This evidence underscores the significance of administering both tests consecutively, as they capture distinct yet complementary facets of anxiety-like behavior. Our study found that both tests indicated increased anxious behavior in the HFD-fed mice compared to SD-fed mice. In our study, OF showed a reduction of anxiety behavior after TG68 administration. The EPM test did not show a significant effect on the capacity to explore the open arms of the EPM structure; however, a significant effect was demonstrated on SAP. SAP (stretch–attend posture) is a behavior observed in rodents, particularly mice, characterized by lowering the back, elongating the body, and moving slowly or remaining still. This behavior is associated with anxiety and is intensified in experimental settings, reflecting a conflict between exploration and anxiety. SAP indicates risk assessment, especially in situations where mice exhibit approach-avoidance tendencies. In classical anxiety tests such as the elevated plus maze (EPM), SAP offers a more comprehensive understanding of drug effects and can capture the complexities of anxiety-related behavior in rodent models [49]. According to the literature, anxiety-like behaviors assessed through EPM and OF are linked to the functioning of the basolateral amygdala (BLA) and the medial prefrontal cortex (mPFC), which are essential neural centers for modulating anxiety and emotion-driven behavior [77] and are the target of obesity-induced neuroinflammation.

Our results highlight that the administration of TG68 partially reversed the diminished expression of *TREM2* in the hypothalamus of high-fat diet (HFD) mice while significantly enhancing the expression of markers associated with the anti-inflammatory and neuroprotective phenotype of microglia, specifically *IGF*, *FGF*, *GDNF*, and *BDNF*. This observation may suggest a potential link between the anxiolytic effects observed behaviorally and the anti-neuroinflammatory effects displayed. The observation of pronounced anti-inflammatory effects associated with a significant metabolic improvement, which followed chronic TG68 administration in the HFD model, prompts considerations for ascertaining in future studies the efficacy of TG68 in animal models of neurodegeneration, such as APP-PS1 mice or TREM2 knockout mice.

## 5. Conclusions

In conclusion, TG68 was demonstrated to improve neuroinflammation and anxiety-like behavior in HFD-fed mice, correlating improved metabolic parameters with reduced anxiety-like behaviors. These preliminary findings align with research linking metabolic homeostasis and mental health, particularly in the context of obesity-related neuroinflammation, which paves the way for future studies to establish a direct causal link between metabolic improvements and reductions in anxiety-like behaviors.

## Figures and Tables

**Table 1:** Primer sequences.

Reference Sequence (RefSeq) RNA	Gene Symbol	Primer Sequences (5’–3’)	
NM_009605	ADIPOQ	(F)	AGATGGCACTCCTGGAGAGAAG
		(R)	ACATAAGCGGCTTCTCCAGGCT
NM_007470	APO D	(F)	GGTGAAGCCAAACAGAGCAACG
		(R)	CAGGAGTACACGAGGGCATAGT
NM_007540	BDNF	(F)	GGCTGACACTTTTGAGCACGTC
		(R)	CTCCAAAGGCACTTGACTGCTG
NM_007860	DIO1	(F)	GTAGGCAAGGTGCTAATGACGC
		(R)	ACTGGATGCTGAAGAAGGTGGG
NM_172119	DIO3	(F)	TGCGTATCAGACGACAACCGTC
		(R)	TGGAAGCCATCAGGTCGGACAA
NM_184052	IGF	(F)	GTGGATGCTCTTCAGTTCGTGTG
		(R)	TCCAGTCTCCTCAGATCACAGC
NM_008361.4	IL 1b	(F)	TGGCCTTCCTGATGAGAGCAT
		(R)	GAAGACACGGATTCCCAGGAA
NM_008355	IL 4	(F)	TCACAGCAACGAAGAACACCA
		(R)	CAGGCATCGAAAAGCCCGAA
NM_031168	IL-6	(F)	TACCACTTCACAAGTCGGAGGC
		(R)	CTGCAAGTGCATCATCGTTGTTC
NM_010548	IL10	(F)	CGGGAAGACAATAACTGCACCC
		(R)	CGGTTAGCAGTATGTTGTCCAGC
NM_008006	FGF 2	(F)	AAGCGGCTCTACTGCAAGAACG
		(R)	CCTTGATAGACACAACTCCTCTC
NM_008084	GAPDH	(F)	ACACCAGTAGACTCCACGACA
		(R)	ACGGCAAATTCAACGGCACAG
NM_011400	GLUT 1	(F)	GCTTCTCCAACTGGACCTCAAAC
		(R)	ACGAGGAGCACCGTGAAGATGA
NM_011401	GLUT 3	(F)	CCGCTTCTCATCTCCATTGTCC
		(R)	CCTGCTCCAATCGTGGCATAGA
NM_019741	GLUT 5	(F)	ATCGCTGCCTTTGGCTCATCCT
		(R)	GCAGCGTCAAGGTGAAGGACT
NM_010438	HMGCoA-R	(F)	CGAGAGGAGGAAGAAAGCAG
		(R)	CGCAGGCTGAGAAATAGAGG
NM_008493	LEPTIN	(F)	AGCCGAGGAGGAGAACAAAG
		(R)	TGACAGGACAGGAGGAGGA
NM_010907	NFkB p65	(F)	CAGAGCAGTACATGGCTTTG
		(R)	CAGCAGGTGGAGGAGGAG
NM_010381	GDNF	(F)	CCTTCGCGCTGACCAGTGACT
		(R)	GCCGCTTGTTTATCTGGTGACC
NM_011146	PPARg	(F)	AGGAGACGACCTGGATTACC
		(R)	AGGCAAGTCCAGAGACATTG
NM_027118	SIRT 6	(F)	GAGAGGAAGGAGTGAGGAG
		(R)	TGGAGTGTAGGAGGAGGAGG
NM_013693	TNFa	(F)	GATGATCCGCGACGTGGAG
		(R)	GAAGTAGACCTGCCCAGTTG
NM_001271100	TREM 2	(F)	GAGCCATCCAGCAGCGT
		(R)	GAGCCCATCCAGCAGCAG

## Data Availability

The original contributions presented in this study are included in the article. Further inquiries can be directed to the corresponding author(s).
